# Fano resonance with high local field enhancement under azimuthally polarized excitation

**DOI:** 10.1038/s41598-017-00785-6

**Published:** 2017-04-21

**Authors:** Wuyun Shang, Fajun Xiao, Weiren Zhu, Hongsen He, Malin Premaratne, Ting Mei, Jianlin Zhao

**Affiliations:** 1 MOE Key Laboratory of Space Applied Physics and Chemistry, and Shaanxi Key Laboratory of Optical Information Technology, School of Science, Northwestern Polytechnical University, Xi’an, 710072 China; 2 Department of Electronic Engineering, Shanghai Jiao Tong University, Shanghai, 200240 China; 3 Advanced Computing and Simulation Laboratory (AχL), Department of Electrical and Computer Systems Engineering, Monash University, Clayton, Victoria 3800 Australia

## Abstract

Being an enabling technology for applications such as ultrasensitive biosensing and surface enhanced spectroscopy, enormous research interests have been focused on further boosting the local field enhancement at Fano resonance. Here, we demonstrate a plasmonic Fano resonance resulting from the interference between a narrow magnetic dipole mode and a broad electric dipole mode in a split-ring resonator (SRR) coupled to a nanoarc structure. Strikingly, when subjected to an azimuthally polarized beam (APB) excitation, the intensity enhancement becomes more than 60 times larger than that for a linearly polarized beam (LPB). We attribute this intensity enhancement to the improved conversion efficiency between the excitation and magnetic dipole mode along with improved near-field coupling. The APB excited Fano structure is further used as a nanoruler and beam misalignment sensor, due to the high sensitivity of intensity enhancement and scattering spectra to structure irregularities and excitation beam misalignment. Interestingly, we find that, regardless of the presence of structural translations, the proposed structure still maintains over 60 times better intensity enhancement under APB excitation compared to LPB excitation. Moreover, even if the APB excitation is somewhat misaligned, our Fano structure still manages to give a larger intensity enhancement than its counterpart excited by LPB.

## Introduction

Localized surface plasmon resonances (LSPRs), resulting from the collective electron oscillations in metal-dielectric interfaces of nanostructures, have attracted tremendous interests recently^1^. This popularity can be mainly attributed to the ability to concentrate and manipulate light in nanoscale regime. One of the most intriguing properties of LSPRs is the strong electromagnetic field enhancement at curved interfaces with small radius of curvature due to strong charge localization. Such strong field enhancements can boost a variety of linear and nonlinear optical processes that can be exploited for practical applications such as nano-optical signal-processing circuits^2^. Plasmonic Fano structures represent one of the celebrated examples that depends on the enhanced field effect^3–5^. The Fano resonances, that can be identified due to their characteristic asymmetric lineshapes were first discovered in atomic systems, experiencing interference between discrete and continuum states^6^. Analogously, Fano resonances in plasmonic structures can be considered as the interference between a narrow dark (subradiant) mode and a broad bright (superradiant) mode via the near-field coupling^7^. So far, Fano resonances have been extensively studied in various plasmonic nanostructures, including dolmen^8, 9^, metallic nanoclusters^10–12^, ring/disk and ring/crescent-ring cavities^13, 14^, core-shell nanostructures^15–17^ and split-ring resonators (SRRs)^18–22^. Owing to plasmonic Fano resonances exhibit pronounced asymmetric spectral lineshapes and highly enhanced local fields, they can be effectively exploited for applications ranging from chemical and biological sensors^23, 24^, to surface enhanced Raman spectroscopy (SERS)^3, 5^ and nonlinear optics^25, 26^.

Local field enhancement at Fano resonance may be of high utility for aforementioned applications. One possibility to boost the intensity enhancement is to change the geometry of the structure. However, if the shape is fixed, one may change the polarization direction of the excitation, noting that such changes could potentially alter the coupling strength of the electric field with the structure. The latter observation is the main focus of this paper. Up to now, it has been demonstrated that cylindrical vector beams (CVBs) can excite a much brighter “hot spot” in plasmonic lens^27–29^, multimers^30–33^ and SRRs^34–37^ than their counterparts produced by linearly polarized beams (LPBs). CVBs exemplified by radially and azimuthally polarized beams (APBs) can be generated and reconfigured by a spatial light modulator (SLM) at ease^38–40^. Therefore, it can provide a much more flexible and effective approach to excite a giant local field by matching the polarization states of the illumination to the eigenmodes of the plasmonic structures.

In this paper, we propose a method to boost the intensity enhancement of a SRR and nanoarc combined Fano structure subjected to APB excitation. In contrast to the conventional excitation with LPB, our scheme can increase the local field intensity by more than 60 times. Moreover, with the excitation of APB, we obtain a 3 times larger local field enhancement for our Fano structure compared to the individual SRR. We further evaluate the performance of the APB excited Fano structure when served as a nanoruler and beam misalignment sensor. Simultaneously, we investigate the APB produced intensity enhancement under the consideration of the structural translations of the Fano structure and the misalignment between excitation beam and structure. Our results provide a new possibility to control the near-field properties of Fano resonance, and could find applications in SERS and biosensing.

## Results and Discussions

**Figure 1:**
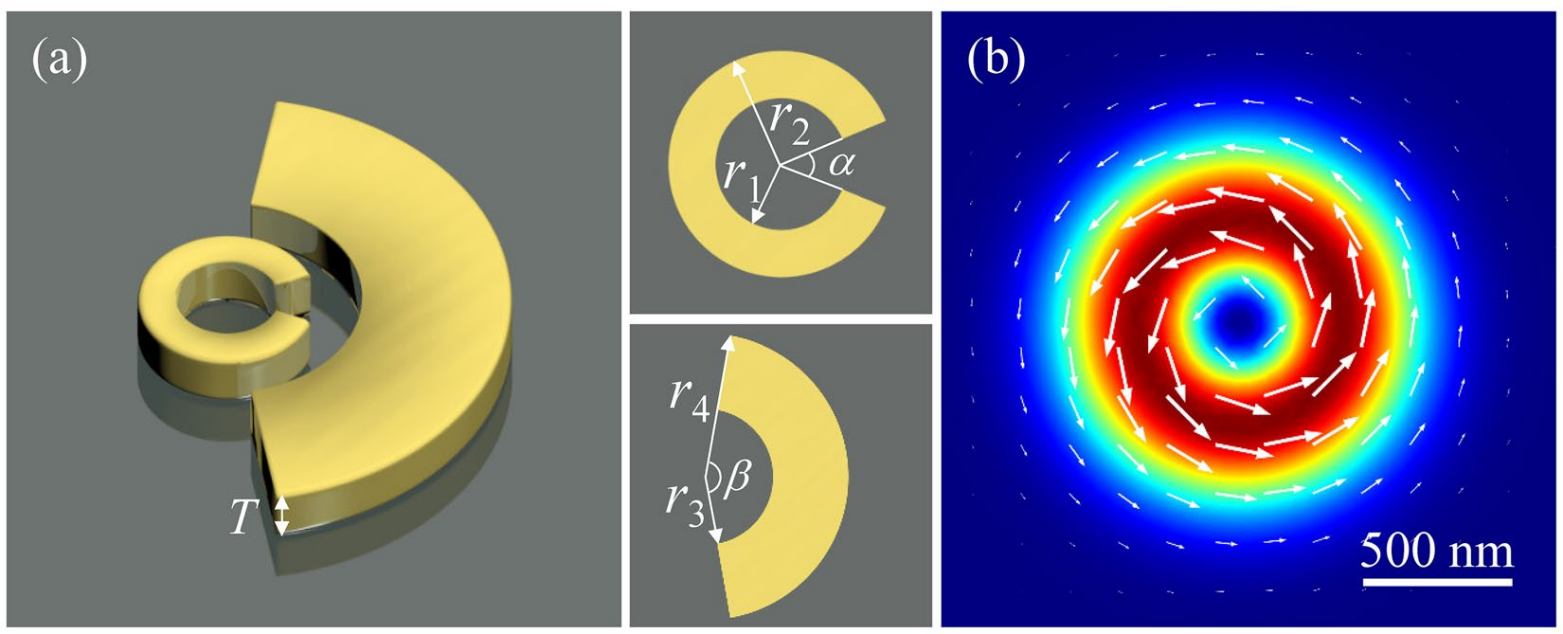
(**a**) Schematic view of the split-ring resonator (SRR) and nanoarc combined structure with the geometry parameters: *r*_1_, *r*_2_, *r*_3_, *r*_4_, *α*, *β* and *T*. (**b**) Intensity and electric vector distributions of an azimuthally polarized beam (APB).

Figure 1(a) shows a schematic diagram of the proposed Fano structure, which is composed of a gold SRR and a nanoarc coaxially. The SRR has an angular gap *α*, inner radius *r*_1_, and outer radius *r*_2_. Similarly, the nanoarc has a central angle *β*, inner radius *r*_3_, and outer radius *r*_4_. The thickness *T* of the structure is kept as a constant (40 nm) throughout this paper. Figure 1(b) displays a typical intensity profile of an APB, which has the polarization direction aligned along the azimuthal direction.

**Figure 2:**
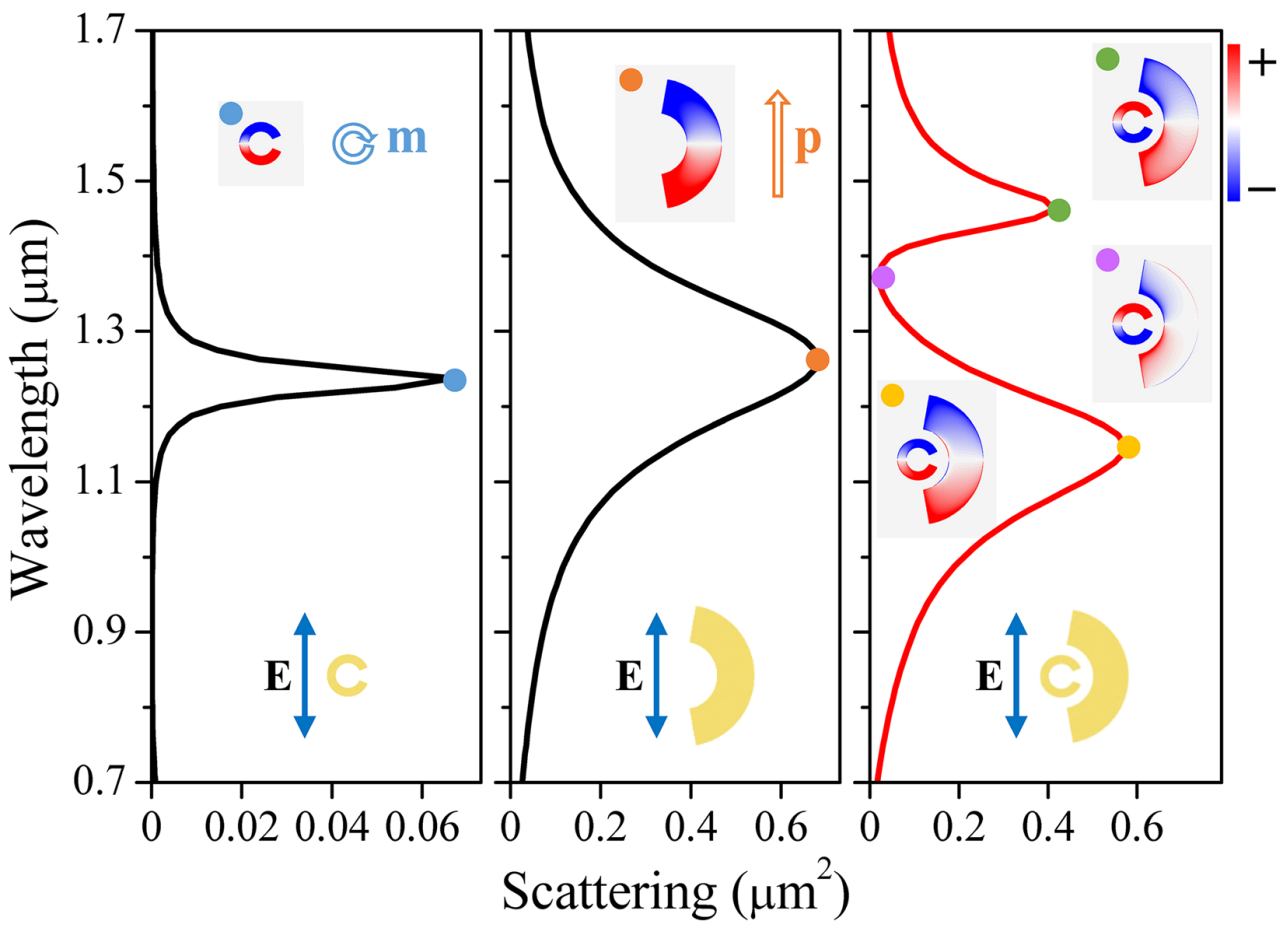
Scattering spectra of SRR (left), nanoarc (middle) and the combined structure (right) with the illumination of linearly polarized beam (LPB). The upper and lower insets are the charge distributions at the labeled wavelengths and the polarizations of the excitation field.

The optical response of the designed structure is numerically calculated by using the finite-difference time-domain (FDTD) method^41^. We start our discussion with scattering spectrum of the SRR-nanoarc structure under the excitation of an LPB at normal incidence, whose polarization is indicated in the lower insets of Fig. 2. Here, geometrical parameters are taken as: *r*_1_ = 35 nm, *r*_2_ = 60 nm, *r*_3_ = 90 nm, *r*_4_ = 190 nm, *α* = 45° and *β* = 160°. Figure 2, from the left to right panels, displays the scattering spectra of the individual SRR, nanoarc, and the combined structure. The scattering spectra of the individual SRR and nanoarc are dominated by a narrow and a broad resonances, respectively. We attribute these two resonances as the magnetic and electric dipole modes^20^, which can be confirmed by their charge distributions in the upper insets of Fig. 2. The magnetic dipole mode experiences weak radiation loss, therefore, it can be considered as a collection of “discrete” states. On the contrary, the electric dipole mode is highly radiative, and thus it is treated as a “continuum” state. The interference between these two modes leads to a pronounced spectral dip located at 1.37 *μ*m in the scattering spectrum of the combined structure, which indicates the occurrence of Fano resonance. The origin of this Fano resonance when interpreted within the framework of hybridization theory^42^, can be attributed to the interference between bonding (~1.45 *μ*m) and anti-bonding modes (~1.15 *μ*m) shown in the right panel of Fig. 2.

**Figure 3:**
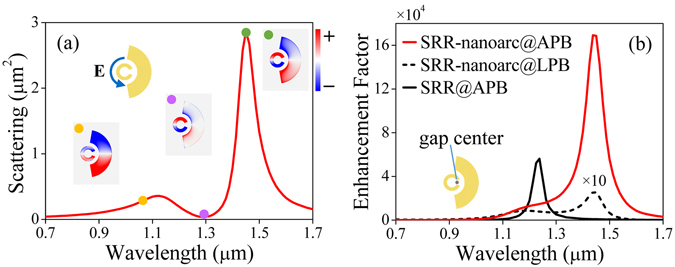
(**a**) Scattering spectrum of the SRR-nanoarc structure for the excitation of APB. The insets indicate the polarization of the excitation field and the charge distributions at the labeled wavelengths. (**b**) Enhancement factor (EF) of the SRR excited by APB (black solid curve) and the SRR-nanoarc structure illuminated by APB (red solid curve) and LPB (black dashed curve), where the EF for the excitation of LPB is multiplied by 10 for clear comparison. The inset indicates the gap center of SRR.

In following analysis, we choose an APB with diameter of 700 nm at normal incidence as the excitation^43^. The red curve in Fig. 3(a) shows the scattering spectrum of the SRR-nanoarc when illuminated by the APB. Different from its counterpart produced by LPB [the red curve in Fig. 2], the APB excited Fano spectral lineshape exhibits a much stronger bonding mode resonance and a weaker anti-bonding mode resonance. Qualitatively, this can be attributed to the matching between the polarization state of APB and plasmonic modes. In term of field distribution, the APB has a better overlap with the azimuthally distributed Fano structure. Therefore, as a driving force, APB can effectively excite and enhance the oscillations along the same azimuthal direction (bonding mode), but decrease the oppositely aligned azimuthal oscillations (anti-bonding mode). As shown in Fig. 3(b), the enhancement factor (EF), defined by the ratio of near-field intensity at the gap center of SRR [as indicated in the insets of Fig. 3(b)] compared to the situation without SRR-nanoarc, is further examined. It is shown that the EF is significantly increased for the SRR-nanoarc structure illuminated by APB [red solid curve in Fig. 3(b)] compared with that illuminated by LPB [black dashed curve in Fig. 3(b)]. Especially, with the excitation of APB, the EF has an over 60 times increase at the wavelength region where the bonding mode exists. In addition, with the excitation of APB, our SRR-nanoarc Fano structure exhibits a 3-fold larger local field enhancement compared to the individual SRR [black solid curve in Fig. 3(b)]. This obvious intensity enhancement effect can be exploited for applications such as ultrasensitive biosensing and SERS.

In order to provide a deeper understanding of our system, a coupled oscillator model is employed to describe the Fano resonance of the SRR-nanoarc structure excited by LPB and APB. The two oscillators provide the anti-bonding and bonding modes, which are characterized by the resonance frequencies *ω*_*a*_, *ω*_*b*_, nonradiative damping efficiencies *γ*_*a*_, *γ*_*b*_ and radiation coupling efficiencies *η*_*a*_, *η*_*b*_. The equation governing the motions of the two oscillators can be written as^26, 44^

1 $$\begin{array}{l}\frac{{d}^{2}{x}_{a}}{d{t}^{2}}+{\gamma }_{a}\frac{d{x}_{a}}{dt}+{\omega }_{a}^{2}{x}_{a}+g{x}_{b}=\frac{1}{2}({\eta }_{b}\frac{{d}^{3}{x}_{b}}{d{t}^{3}}+{\eta }_{a}\frac{{d}^{3}{x}_{a}}{d{t}^{3}})+{\eta }_{a}E\\ \frac{{d}^{2}{x}_{b}}{d{t}^{2}}+{\gamma }_{b}\frac{d{x}_{b}}{dt}+{\omega }_{b}^{2}{x}_{b}+g{x}_{a}=\frac{1}{2}({\eta }_{b}\frac{{d}^{3}{x}_{b}}{d{t}^{3}}+{\eta }_{a}\frac{{d}^{3}{x}_{a}}{d{t}^{3}})+{\eta }_{b}E\end{array},$$

where *g* denotes the coupling constant between the two oscillators and *E* = *E*_0_e^i*ωt*^ represents the electric field of the excitation beam. At steady state, the motions of two oscillators are harmonics with forms of *x*_*a*,*b*_ = *C*_*a*,*b*_e^i*ωt*^, where *C*_*a*,*b*_ can be derived as

2 $$\begin{array}{rcl}{C}_{a}(\omega ) & = & \frac{(g+\frac{i}{2}{\eta }_{b}{\omega }^{3}){C}_{b}(\omega )-{\eta }_{a}{E}_{0}}{{\omega }^{2}-i{\gamma }_{a}\omega -{\omega }_{a}^{2}-\frac{i}{2}{\eta }_{a}{\omega }^{3}}\\ {C}_{b}(\omega ) & = & \frac{-{\eta }_{a}{E}_{0}(g+\frac{i}{2}{\eta }_{a}{\omega }^{3})-{\eta }_{b}{E}_{0}({\omega }^{2}-i{\gamma }_{a}\omega -{\omega }_{a}^{2}-\frac{i}{2}{\eta }_{a}{\omega }^{3})}{({\omega }^{2}-i{\gamma }_{b}\omega -{\omega }_{b}^{2}-\frac{i}{2}{\eta }_{b}{\omega }^{3})({\omega }^{2}-i{\gamma }_{a}\omega -{\omega }_{a}^{2}-\frac{i}{2}{\eta }_{a}{\omega }^{3})-(g+\frac{i}{2}{\eta }_{b}{\omega }^{3})(g+\frac{i}{2}{\eta }_{a}{\omega }^{3})}.\end{array}$$

**Figure 4:**
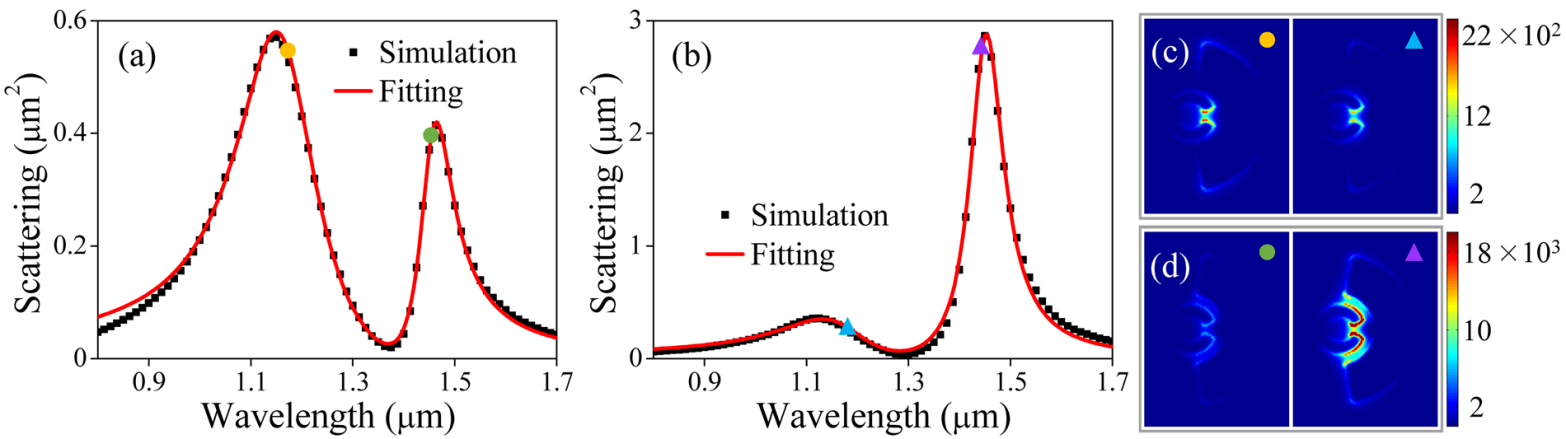
Scattering spectra of SRR-nanoarc structure excited by (**a**) LPB and (**b**) APB, where the black dots and red solid curves are the simulation and fitting results, respectively. The intensity distributions of the corresponding (**c**) anti-bonding and (**d**) bonding modes under the excitation of LPB (left) and APB (right), respectively.

The scattering coefficient of the system can be described by *σ*_*sca*_ = *I*_0_ + |*C*_*a*_ + *C*_*b*_|^2^ with *I*_0_ accounting for the background, which is used to fit the scattering spectra excited by LPB and APB with the same excitation power over the structure. A good agreement between the theory (red solid curves) and simulation (black dots) is achieved, as depicted in Fig. 4(a) and (b). The fitting parameters are listed in Table 1, from which two emerging features are found. (1) There is an increase in *η*_*b*_ under the excitation of APB, which suggests that the APB has a better coupling with the bonding mode. This results in an enhanced scattering in long wavelength regime, exhibiting a more profound asymmetric spectral lineshape as shown in Fig. 4(b). (2) A significant increase in coupling constant *g* is found in our system with the excitation of APB. This leads to the enhanced near-field intensity of the SRR-nanoarc structure, which can be confirmed by comparing the near-field intensity distributions at the top surface of the structure with LPB and APB illumination [see Fig. 4(c) and (d)].

**Table 1:** Extracted fitting parameters for Fano resonance excited by LPB and APB.

	*ω*_*a*_	*ω*_*b*_	*γ*_*a*_	*γ*_*b*_	*g*	*η*_*a*_	*η*_*b*_	*I*_0_	*E*_0_
**LPB**	1.171	1.454	0.174	0.065	0.06	0.034	0.009	0	5.52
**APB**	1.183	1.448	0.197	0.04	0.115	0.031	0.028	0	

**Figure 5:**
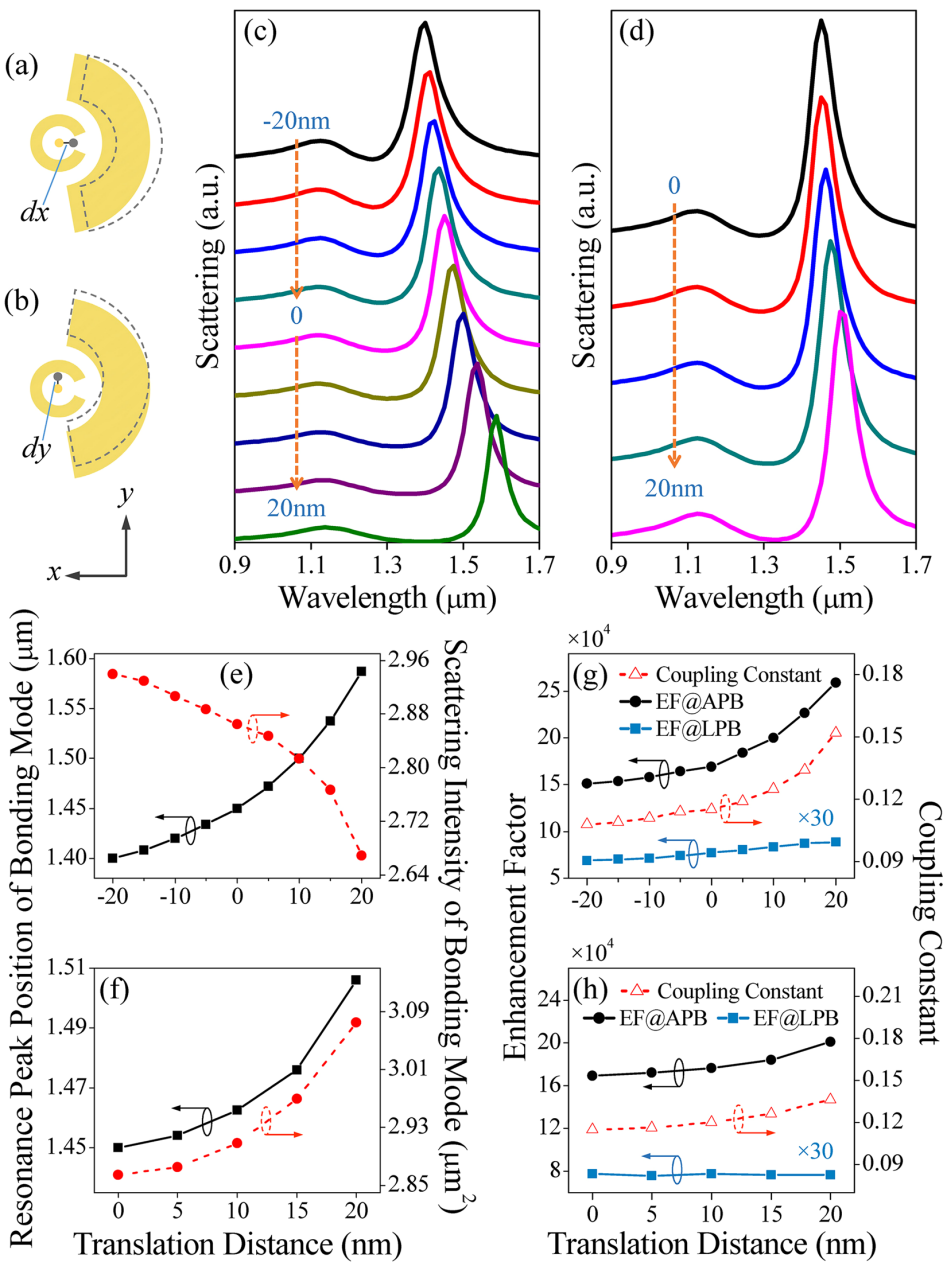
Schematic of the in-plane translation of the SRR-nanoarc structure along (**a**) *x* and (**b**) *y* directions, where the translations of nanoarc are indicated by gray dashed lines. Scattering spectra of SRR-nanoarc structure under the structural translations along (**c**) *x* and (**d**) *y* directions. Resonance peak position (black rectangles) and scattering intensity (red dots) of bonding mode with APB excitation depend on the nanoarc displacement along (**e**) *x* and (**f**) *y* directions. EF (black dots) and coupling constant (red triangles) with APB excitation versus the nanoarc displacement along (**g**) *x* and (**h**) *y* directions. Here, the rectangles are the LPB excited EF, which is magnified by 30 for comparison.

Owing to the enhanced scattering and more profound asymmetric spectral lineshape obtained from the Fano structure when illuminated by APB, it becomes much easier to detect and characterize the spectrum signal in practical applications. In the following, we demonstrate our system can serve as a nanoruler, which is important for determining nanoscale distances within chemical or biological species^45, 46^. Here, the APB excitation is kept coaxially with the SRR and the nanoarc has an in-plane displacement along *x* and *y* directions as illustrated in Fig. 5(a) and (b). Figure 5(c) and (d) show the scattering spectra of SRR-nanoarc structure with the translations along *x* direction from −20 nm to 20 nm and *y* direction from 0 nm to 20 nm, respectively. It can be found that the SRR-nanoarc structure causes an obvious spectral lineshape shifting even down to the nanometer scale of structural translation. The resonance peak position and scattering intensity of bonding mode depend on in-plane displacement are depicted in Fig. 5(e) and (f), which allow us to evaluate the magnitudes as well as the directions of structural translations. Such high sensitivity to structural translations results from the SRR-nanoarc gap dependent coupling strength, which is exponentially reduced when gap size increases [the red triangles in Fig. 5(g) and (h)], decreasing the spectral overlapping between the bonding and anti-bonding modes. It also suggests that the variation of coupling strength dominates the change on intensity EF shown as black dots in Fig. 5(g) and (h), which correspond to EF for the structural translations along the *x* and *y* directions, respectively. Interestingly, despite having imperfections in geometry, the APB excited SRR-nanoarc structure exhibits an EF that is regularly over 60 times larger than its LPB excited counterpart [the blue rectangles in Fig. 5(g) and (h)], and robustly maintains more than 1.5 × 10^5^.

**Figure 6:**
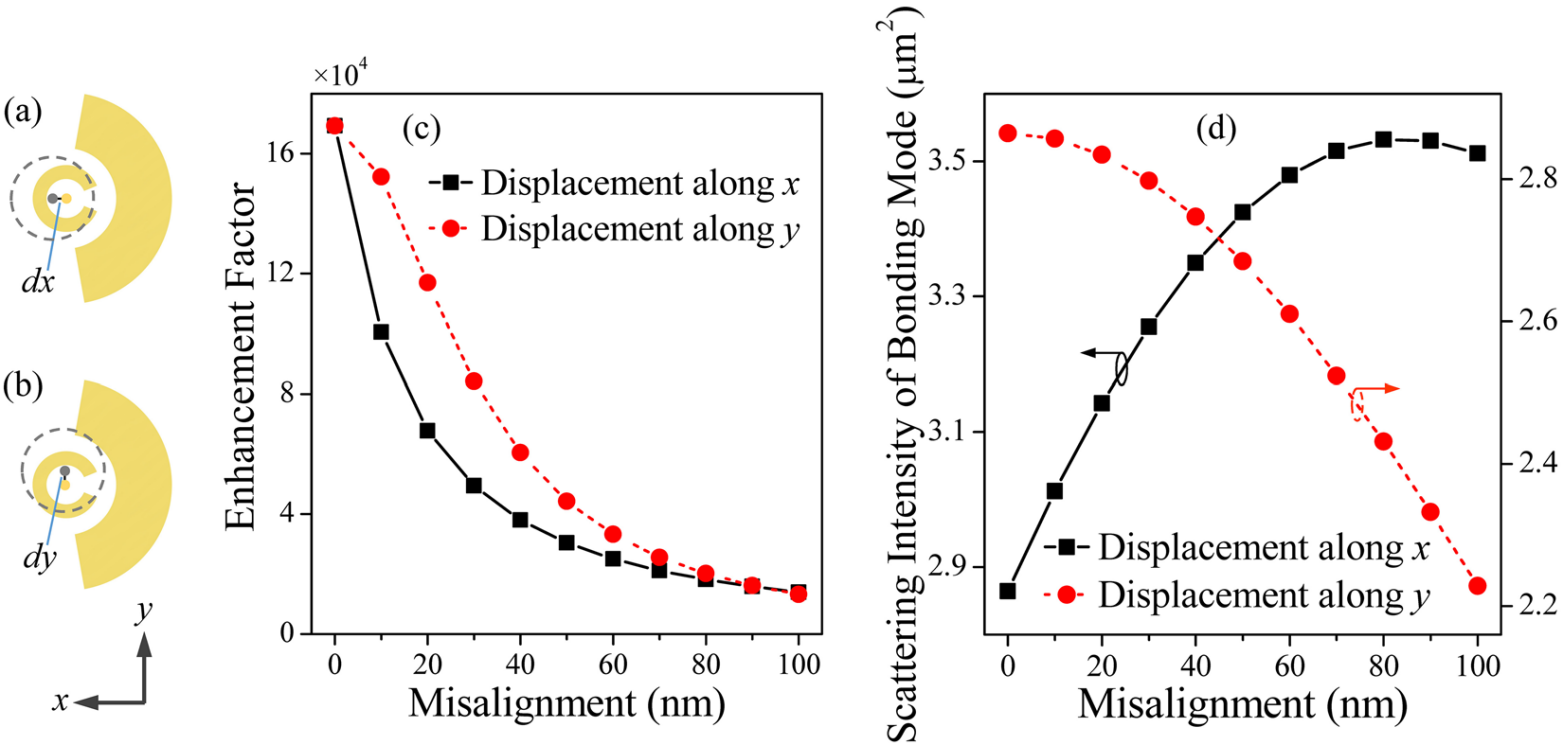
Schematic of the in-plane misalignment between APB and SRR-nanoarc structure along (**a**) *x* and (**b**) *y* directions, where the positions of APB are indicated by gray dashed circles. (**c**) EF and (**d**) scattering intensity of bonding mode versus the displacements of APB along *x* (black rectangles) and *y* (red dots) directions.

Apparently, the maximum near-field intensity enhancement is obtained for coaxial overlap between the APB and SRR-nanoarc structure. However, such registration is quite difficult in nanoscale regime and some misalignment might occur in practice. In order to study the impact of intensity enhancement subjected to the in-plane misalignment between excitation beam and structure, displacements of APB along *x* and *y* directions are taken into account, as depicted in Fig. 6(a) and (b). The EF decreases as APB has a displacement from the SRR-nanoarc as shown in Fig. 6(c). Nevertheless, intensity enhancement of SRR-nanoarc under APB is more than 5 times larger than its LPB excited counterpart even for a misalignment of 100 nm (over half the outer radius of nanoarc) between APB and the structure. In addition to this high sensitivity of intensity EF to the displacement between excitation beam and center of the structure, scattering intensity of bonding mode is also changed when the beam is misaligned, as illustrated in Fig. 6(d). The scattering intensities change inversely as APB transforms along *x* and *y* directions. According to the near-field intensity enhancement and far-field scattering spectra, our system can potentially serve as a beam misalignment sensor.

## Conclusion

In summary, we have demonstrated the Fano resonance in a gold SRR-nanoarc structure. Our results show that, compared with LPB illumination, a much more asymmetric Fano lineshape is obtained under APB excitation. Moreover, the APB excited Fano resonance shows a more than 60 times larger intensity EF than its counterpart excited by LPB. Surprisingly, compared to the LPB, APB can produce a much stronger intensity enhancement even if the Fano structure is subjected to in-plane translation deviations and excitation beam is misaligned. Our results may find applications in nanoscale distances measurement, surface enhanced spectroscopy and ultrasensitive biosensing.

## Methods

### Simulation

The transverse electric field of the APB can be expressed as^39^

3 $${{\bf{E}}}_{\varphi }=H{G}_{01}{{\bf{e}}}_{x}+H{G}_{10}{{\bf{e}}}_{y},$$

where *HG*_01_ and *HG*_10_ represent two orthogonally polarized Hermite-Gauss modes with the unit vectors **e**_*x*_ and **e**_*y*_, respectively. The Hermite-Gauss mode can be described as^39^

4 $$H{G}_{mn}(x,y,z)={E}_{0}{H}_{m}[\sqrt{2}\frac{x}{w(z)}]{H}_{n}[\sqrt{2}\frac{y}{w(z)}]\frac{{w}_{0}}{w(z)}\exp [-i{\phi }_{mn}(z)]\exp [i\frac{k{r}^{2}}{2q(z)}]\mathrm{.}$$

Here, *H*_*m*_ and *H*_*n*_ denote the Hermite polynomials, *E*_0_ is the constant electric field amplitude, $w(z)=\{{w}_{0}^{2}/\{1+{\mathrm{[2}z/(k{w}_{0}^{2})]}^{2}{\}\}}^{\mathrm{1/2}}$ is the beam size with the beam waist *w*_0_ = *w*(0), ${z}_{0}=(\pi {w}_{0}^{2})/\lambda$ is the Rayleigh range, *q*(*z*) = *z* − *iz*_0_ is the complex beam parameter, *φ*_*mn*_ = (*m* + *n* + 1) tan^−1^(*z*/*z*_0_) is the Gouy phase shift, *r* is the radial coordinate and *k* = 2*π*/*λ* is the wavenumber. According to Eq. (4), we define *d*_*max*_ = 2(*z*_0_/*k*)^1/2^, which refers to the diameter of the maximum intensity circle of the incident field, to characterize the beam size of APB^36^.

In our FDTD simulations, perfectly matched boundaries are used to mimic single nanostructure in infinitely large free space. The nanostructure is swept-meshed with the minimum size of 5 nm. The structure is immerged in air with the permittivity of gold taken from ref. 47 and fitted to the Drude model in the visible and near-infrared regions.
